# The “Aachen fall prevention App” – a Smartphone application app for the self-assessment of elderly patients at risk for ground level falls

**DOI:** 10.1186/s13037-017-0130-4

**Published:** 2017-05-08

**Authors:** Peter Rasche, Alexander Mertens, Christina Bröhl, Sabine Theis, Tobias Seinsch, Matthias Wille, Hans-Christoph Pape, Matthias Knobe

**Affiliations:** 1 Chair and Institute of Industrial Engineering and Ergonomics of RWTH Aachen, Bergdriesch 27, 52072 Aachen, Germany; 2 Division of Orthopaedic Trauma, Department of Surgery, University Hospital Zurich, 8091 Zurich, Switzerland; 3 Department of Orthopaedic Trauma, University of Aachen Medical Center, Pauwelsstraße 30, 52074 Aachen, Germany

**Keywords:** Orthogeriatrics, Co-managed care, Fracture in the elderly, Geriatric trauma center, Fall prevention, Balance assessment, mHealth

## Abstract

**Background:**

Fall incidents are a major problem for patients and healthcare. The “Aachen Fall Prevention App” (AFPA) represents the first mobile Health (mHealth) application (app) empowering older patients (persons 50+ years) to self-assess and monitor their individual fall risk. Self-assessment is based on the “Aachen Fall Prevention Scale,” which consists of three steps. First, patients answer ten standardized yes–no questions (positive criterion ≥ 5 “Yes” responses). Second, a ten-second test of free standing without compensatory movement is performed (positive criterion: compensatory movement). Finally, during the third step, patients rate their subjective fall risk on a 10-point Likert scale, based on the results of steps one and two. The purpose of this app is (1) to offer a low-threshold service through which individuals can independently monitor their individual fall risk and (2) to collect data about how a patient-centered mHealth app for fall risk assessment is used in the field.

**Results:**

The results represent the first year of an ongoing field study. From December 2015 to December 2016, 197 persons downloaded the AFPA (iOS^™^ and Android^™^; free of charge). *N* = 111 of these persons voluntarily shared their data and thereby participated in the field study. Data from a final number of *n* = 79 persons were analyzed due to exclusion criteria (age, missing objective fall risk, missing self-assessment). The objective fall risk and the self-assessed subjective risk measured by the AFPA showed a significant positive relationship.

**Conclusions:**

The “Aachen Fall Prevention App” (AFPA) is an mHealth app released for iOS and Android. This field study revealed the AFPA as a promising tool to raise older adults’ awareness of their individual fall risk by means of a low-threshold patient-driven fall risk assessment tool.

## Background

The incidence of falls in the older adult population is difficult to determine, but the consequences of falls are a major public health and economic issue [1–4]. About 30% of community-dwelling people older than 65 years fall at least once a year [5]. Some evidence indicates that falls can be prevented [1]; therefore, the risk of falling needs to be identified and monitored, starting at an early age (50+ years). At present, patients have to consult their physician or a hospital for risk assessment, as the usual screening algorithms rely on trained individuals in a hospital setting [1]. Consequently, assessing the fall risk is not a low threshold service, so empowering patients with an independent pre-test of their fall risk by a home-based screening method would have considerable advantages. This method should be distinct from the more intensive assessment procedures currently used to identify potentially modifiable risk factors in multifactorial fall prevention programs [6]. A simple self-assessment approach would seem useful for monitoring individual fall risk in the first place and, in cases indicating an increased risk, a specialist could perform a clinical fall risk assessment [7]. Studies have shown that a simple balance test is a quite good indicator of a specific fall risk [8–11]. Simple screening questions have also been identified to perform as well as more complex screening tests in predicting those who will fall [6]. The “Aachen Fall Prevention App” (AFPA) combines these ideas, based on the “Aachen Fall Prevention Scale” [7].

## Method

### Design

A field study is ongoing, but this article includes the results from the first year of observation (December 2015 to December 2016). The overall aim of the field study is to determine whether a mobile health app offering self-assessment of fall risk would gain interest and would be used if just presented in the major app stores of Google and Apple. Furthermore, the collected data should reveal whether a suitable relationship exists between an objective clinical and a subjective self-assessed fall risk, as measured by the AFPA.

### Measuring subjective fall risk

**Fig. 1:**
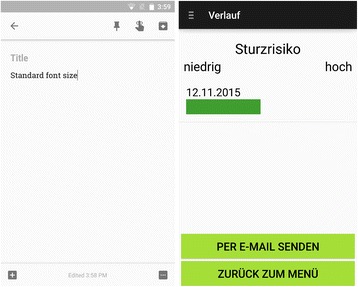
Comparison between a classical Android App and ‘Aachen Falls Prevention App’ (screenshot, Android V4.0.3. January 2016; “Verlauf” = overview, “Sturzrisiko” = fall risk, “niedrig” = low, “hoch” = high, “per E-Mail senden” = send via e-mail, “Zurück zum Menü” = back to menu)

Subjective fall risk was measured using the newly developed AFPA, which enables users to self-assess their fall risk using a three-step self-assessment based on the “Aachen Falls Prevention Scale” [7]. First, the patients perform a self-test containing ten standardized yes–no questions (positive criterion ≥ 5 “yes” responses). Second, a balance test of ten seconds of free standing without compensatory movement was performed (positive criterion: compensatory movement). Based on the results of step one and two, patients rated their subjective fall risk on a 10-point Likert scale (positive criterion ≥ 5 points) as the third step. To reach as many users as possible, the app was developed for both iOS and Android. Furthermore, the age-related limitations of the targeted user population (persons 50+ years) were considered by choosing suitable font sizes, high-contrast interfaces, and a fully usable demo-mode to train in the app use without the fear of failing [12]. Figure 1 shows a comparison of a typical interface according to Android guidelines and that of the age-responsively designed AFPA (Fig. 1). Additionally, a reminder function was included within the app, as the self-assessment should be repeated regularly every three months. Users could also independently share self-assessment data with a trusted person via e-mail.

### Measuring objective fall risk

The number of falls within the past year is a good indicator of an objective fall risk [13]. Therefore, participants taking part in the field study were asked, via the AFPA, whether they had fallen within the past year (0 falls; 1 to 3 falls; more than 3 falls).

### Data collection

The AFPA was released in December 2015. In this article, the analyzed data were collected from December 2015 to December 2016. No specific recruitment of app users was performed for ecological validity. The aim was to determine whether a prevention app would be accepted and downloaded voluntarily by persons in the target population. Therefore, the app is still available for gathering further data. Users are able to decide whether they would like to use the app privately without sharing data for this field study or not. Shared data include demographics (age, gender and measured objective falls’ risk), self-assessment results and app use data like number of performed self-assessments.

### Statistics

Data were analyzed using the SPSS statistics software, version SPSS 22 (IBM). Several one-way analyses of variance (ANOVA) were conducted at a significance level of .05.

### Participants

The AFPA was downloaded a total of 197 times. A total of *n* = 111 persons participated in the field study. Inclusion criteria for analysis were age greater than 50 years, a clear statement of the objective fall risk, and at least one self-assessment of subjective falls. A total of *n* = 79 persons met these criteria and were included in the analysis. The mean age within this sample was 63 years, with an age range from 50 to 70 years. Gender was just mentioned by 16 of the 79 participants (1 male, 15 female). In total, 89% (*n* = 70) participants used the iOS version of the AFPA.

## Results

Objective fall risk was identified for 20% (*n* = 16) of the participants. During self-test participants answered, on average, 1.35 of the standardized questions with “yes.” The most frequent positively answered question was whether participants used a walking aid. For 13 participants, a positive criterion with more than five “yes” answers was measured during the first step of self-assessment. The ten-second free-standing test was failed by five participants. The third step subjective fall risk rating had an average of 0.7 points (SD = 1.9 points) on the 10-point Likert scale (0 points = low risk; 10 points = high risk).

**Fig. 2:**
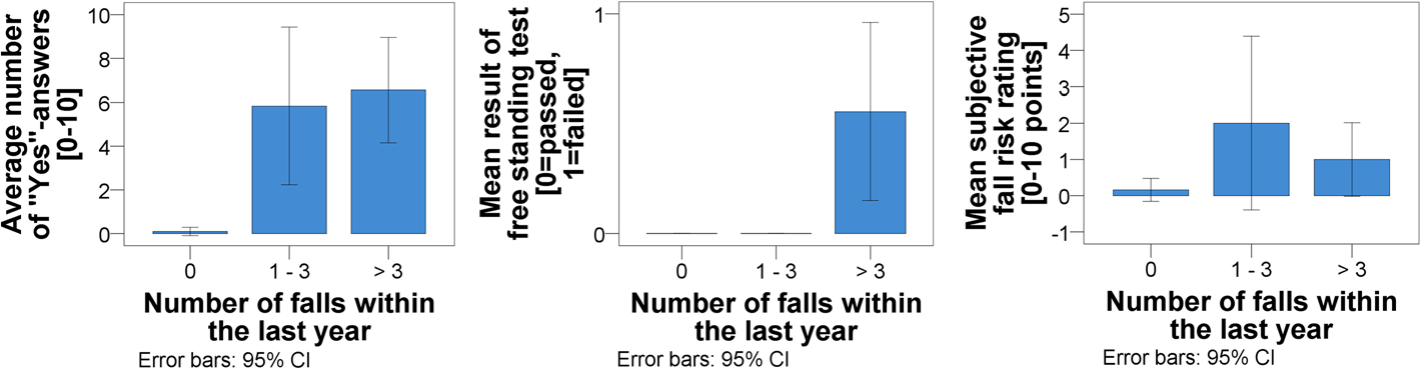
Descriptive data for the one-way analyses of variance regarding objective fall risk

The objective fall risk significantly corresponded with the number of registered “yes” answers per participant for the ten standardized questions, F(1, 75) = 100.73, *p* <0,001, η^2^ = 0.729. Participants with an objective fall risk answered more questions with “yes” during self-test than did the participants without an objective fall risk (positive criterion ≥ 5 “yes” responses; see Fig. 2). Furthermore, a significant positive relationship was revealed between the objective fall risk and failure in the ten-second free-standing test, F(2, 75) = 32.692, *p* <0.001, η^2^ = 0.525. Participants with an objective fall risk failed the free-standing test more often (Fig. 2). Finally, the third one-way analyses of variance identified a significant positive relationship between the objective fall risk and the subjective fall risk rating, F(2, 75) = 6.033, *p* = 0.004, η^2^ = 0.139. Participants with an objective fall risk rated their subjective fall risk as higher (Fig. 2).

The average number of times the participants performed the self-assessment was 1.7 (SD = 1.2) times, ranging from one time up to nine times per participant. No deviation of fall risk over repeated measurement was detected.

## Discussion

A significant relationship was identified between the objective fall risk and the self-assessment results obtained by the AFPA. Participants with a high objective fall risk also showed a high risk as measured by the AFPA. In the past, time-consuming clinical measurement sets showed shortcomings in discrimination between fallers and non-fallers based on a self-reported retrospective falls-status [14]. Therefore, this study was able to show the potential of mobile Health apps in the context of patient-driven fall risk assessment.

Clinical fall risk assessment usually starts at an age of 75 years [4, 6–11]. The AFPA was downloaded voluntarily without specific recruitment by even younger participants, indicating a specific interest in this topic among older adults who use information and communication technology [15]. Hence, the app seems to be a suitable medium for offering a simple and low-threshold service for fall risk assessment in older adults. Nevertheless, the results of the first year of field study revealed a short duration of usage. Participants assessed their fall risk about two times, on average. Further work is needed to extend the capabilities of the study app to provide more than just self-assessment by offering suitable arrangements to support users’ self-paced prevention measures. Possible features might include guided instructions to strengthen power and balance. Further studies are also needed to investigate which incentives could be facilitated to increase adherence, as discussed in the context of health-related exergames [16]. Exergames run on mobile phones and the player is required to be physically active [16]. Medical and public health communities have discussed the potential of these games with regard to their influence on higher levels of sustainable physical activity to achieve health benefits [16]. Nevertheless, this study showed the potential of modern digital technology in the context of patient-driven fall risk assessment.

## Limitations

This study has certain limitations which should be mentioned. First the “Aachen Falls Prevention Scale” is still under evaluation and validation in the laboratory setting. Nevertheless, the results of this study indicate a suitable validity, as significant relationships were detected between the objective fall risk and the subjective risk measured by the AFPA.

## Conclusion

Assessing patients’ fall risk and providing acceptable preventive measures remains an important research topic. We introduced the “Aachen Falls Prevention App” and presented initial results of the first year of an ongoing field study that recruits participants via the major app-stores of Apple and Google. The results show that this app is a useful supplement in healthcare, as it is a low-threshold service that supports patients in self-assessing their individual fall risk. Collection of more data over the next years will provide more insights for incorporating mHealth solutions into fall prevention and for getting patients initially involved in monitoring their individual fall risks.
